# Objectively-Measured Neighbourhood Attributes as Correlates and Moderators of Quality of Life in Older Adults with Different Living Arrangements: The ALECS Cross-Sectional Study

**DOI:** 10.3390/ijerph16050876

**Published:** 2019-03-10

**Authors:** Casper J. P. Zhang, Anthony Barnett, Janice M. Johnston, Poh-chin Lai, Ruby S. Y. Lee, Cindy H. P. Sit, Ester Cerin

**Affiliations:** 1 School of Public Health, The University of Hong Kong, Hong Kong, China; casperz1@connect.hku.hk (C.J.P.Z.); jjohnsto@hku.hk (J.M.J.); 2 Mary MacKillop Institute for Health Research, Australian Catholic University, Melbourne, Victoria 3000, Australia; Anthony.Barnett@acu.edu.au; 3 Department of Geography, Faculty of Social Sciences, The University of Hong Kong, Hong Kong, China; pclai@hku.hk; 4 Elderly Health Service, Department of Health, The Government of Hong Kong Special Administration Region, Hong Kong, China; ruby_sy_lee@dh.gov.hk; 5 Department of Sports Science and Physical Education, Faculty of Education, The Chinese University of Hong Kong, Hong Kong, China; sithp@cuhk.edu.hk

**Keywords:** geographic information systems, environmental audits, living arrangements, mega-city, walkability, Hong Kong, social support, mental health

## Abstract

With an ageing world population, preservation of older adults’ health and quality of life (QoL) is paramount. Due to lower levels of physical functionality, older adults are particularly susceptible to local environment influences, especially those living alone and lacking family support. Using generalised additive mixed models, we examined associations and confounder-adjusted associations between objectively-measured neighbourhood attributes and QoL domains in 909 Hong Kong Chinese elderly community dwellers. Most examined neighbourhood attributes were not associated with QoL in the whole sample. Neighbourhood residential and entertainment density was curvilinearly and/or linearly related to specific QoL domains. Number of parks was negatively associated with social QoL and having well-treed parks with higher levels of social QoL. Older adults living alone in neighbourhoods with poor access to destinations and few activities in parks showed lower environmental and/or social QoL than their counterparts. Neighbourhood built environment characteristics do not seem to impact Hong Kong older adults’ physical and psychological QoL. Medium-to-high density, well-ordered neighbourhoods with optimal mixes of well-treed public open spaces and services meeting their daily needs may significantly contribute to social and environmental QoL in this population and appear particularly important to those living alone.

## 1. Introduction

According to the World Health Organisation (WHO), quality of life (QoL) refers to people’s perceptions of their status in life in relation to their goals, standards and concerns, and within the context of their culture and value systems. This concept spans a wide range of aspects of life, including and beyond mere physical and mental health defined as the presence and severity of diseases [1]. The breadth of the WHO’s definition of QoL is reflected in its domain-based QoL scales, the WHOQOL-100 and WHOQOL-BREF, that capture a person’s physical health, psychological state, social relations and satisfaction with essential features of the proximal and distal environment [1].

With extended life expectancy, an increasing proportion of the world population is likely to experience poorer QoL due to age-related chronic health problems and declines in physical functioning and mobility [2]. It is, thus, important to identify large-scale modifiable factors that can help older adults preserve good health and QoL. Among various factors, the physical characteristics of the neighbourhood environment in which older adults live are deemed to influence all domains of QoL [3]. Older adults’ lower levels of physical functionality and mobility make them particularly susceptive to the influence of their local environment. For instance, a well-maintained pedestrian infrastructure can facilitate older people’s engagement in recreational and utilitarian walking, which, in turn, may benefit their physical health and help them maintain their independence [4]. Access to diverse destinations in the neighbourhood may provide opportunities for social interaction, which is essential for psychological QoL [5,6]. These environmental features can also contribute to older adults’ satisfaction with their neighbourhood environment [7]. On the other hand, adverse neighbourhood attributes, such as noise and physical barriers to walking, may have negative effects on QoL [8].

Several studies have found associations between physical attributes of the neighbourhood environment and QoL in older adults. For example, various aspects of neighbourhood safety and quality were positively associated with health-related QoL [9,10]. Higher levels of perceived access to services, shops and public transport were predictive of higher physical and social QoL [9]. Older adults living in neighbourhoods with safe and aesthetically-pleasing parks reported higher levels of physical and psychological QoL [3,11], and spending more time in green areas was found to benefit mental well-being [12].

Most of the studies examining physical environmental correlates of QoL used self-report measures of environmental attributes [3]. Among the handful of studies that used objective measures of the environment [3,13,14,15,16,17], half of them employed a single composite index or variable [14,15,16], while the others examined two to four characteristics. The fact that most studies in this field relied on self-report measures of the environment is problematic because these, in this particular context, are associated with a higher risk of reverse causality than their objective counterparts. Specifically, older adults with better physical and psychological QoL may perceive their neighbourhood environment more positively than their counterparts because their affective states are more positive [18,19,20] and/or they are more mobile and capable of negotiating environmental barriers [21]. Additionally, affective states have been shown to influence perceptions of distance to destinations and slope steepness, whereby, for example, sadder and more fatigued people perceived hills to be steeper and destination distances to be greater than happier and less fatigue people [22]. These findings suggest that people’s QoL and accompanying affective states can influence their perceptions of the neighbourhood environment gauged by self-report measures. Therefore, the use of objective measures to quantify environmental attributes, such as geographic information systems (GIS) [23] or environmental audits conducted by independent assessors [24], can plausibly provide more robust evidence of potential causal effects of the neighbourhood environment on QoL than self-report measures, although, understandably, they cannot not address neighbourhood self-selection bias within the context of observational studies.

Social-ecological models posit that health and well-being outcomes are shaped by the interaction of multiple levels of influences, including environmental, social and individual factors [25]. In the context of these theoretical models, attributes of the neighbourhood environment may directly impact on QoL and/or moderate the effects of other factors on QoL [25]. Among the many key contributors to QoL in later life that may be moderated by attributes of the neighbourhood environment are living arrangements, which represent a micro-environmental (household composition) as well as a social (proxy for access to social contacts and support) factor. There is substantial evidence that, compared to those living with others, older adults living alone tend to receive less personal assistance and emotional support, which results in more negative psychological states [20] and QoL perceptions [26]. In studies conducted in Hong Kong, older adults living alone reported poorer QoL and self-rated health status, limited social networks of relatives, lack of emotional and instrumental support [27] and more depressive symptoms [20,27]. Given the increasing prevalence of older urban dwellers living alone in Western [28] as well as Asian cities, such as Hong Kong [29], it is important to identify modifiable factors that can mitigate the negative effects of living arrangements on older adults’ well-being. It is plausible to assume that living in a neighbourhood that provides good access to health-related services and opportunities for engagement in various social and health-enhancing activities may buffer the negative effect of living alone on QoL [30]. To our knowledge, no studies have examined the moderating effects of the neighbourhood environment on the association between living arrangements and QoL.

Our study aimed to examine the associations between objectively-measured neighbourhood physical environmental attributes and QoL domains in Hong Kong older community dwellers and estimate the moderating effects of neighbourhood environmental attributes on the associations of living arrangements with QoL. We hypothesised that: (1) objective measures of availability of/access to destinations, public open spaces/parks and pedestrian-friendly infrastructure would be positively associated with QoL domains; (2) adverse environmental attributes, such as pollution and traffic-related hazards, would be negatively associated with QoL domains; (3) better access to/availability of destinations and lower levels of adverse environmental attributes would mitigate the negative effect of living alone on QoL.

## 2. Materials and Methods

### 2.1. Study Design and Neighbourhood Selection

This study used data from the Active Lifestyle and the Environment in Chinese Seniors (ALECS) project, an observational investigation of associations between neighbourhood environment, physical activity, depressive symptoms and QoL in Hong Kong Chinese elderly community dwellers. The ALECS project used a two-stage sampling method to recruit participants from 124 tertiary planning units (TPUs) stratified by high/low socio-economic status (SES) and walkability to maximise the variation in environmental characteristics. TPUs are the smallest administrative area units with census data in Hong Kong. TPU-level SES was defined using Census data on median household income. TPUs were classified into high and low walkable based on a walkability index consisting of the sum of TPU-level z-scores for net residential density, intersection density and land-use mix derived using GIS data. Details about study design and neighbourhood selection have been reported elsewhere [31]. Ethics approval for the conduct of this study was obtained by the Department of Health of Hong Kong SAR and The University of Hong Kong Human Research Ethics Committee for Non-Clinical Faculties (ethics approval no.: EA270211; 22 February 2011).

### 2.2. Participants

Participants were recruited from the Elderly Health Centres (EHCs) and elderly community centres located in the pre-selected TPUs. The EHCs were established by the Department of Health of the Hong Kong Special Administrative Region to provide healthcare services to persons aged 65+ years and are distributed across all 18 Hong Kong districts. Although EHC clients are representative of the general Hong Kong elderly population in terms of age and SES, they tend to be more health-conscious than their counterparts [32]. To examine potential bias (better health-related QoL) associated with being a client of the EHCs, approximately 30% participants were recruited from elderly community centres with no formal provision of medical and health services. No significant differences between participants from the two types of recruitment centres were observed in age, physical health, marital status, living arrangements, type of neighbourhood of residence, type of housing, car in the household and health-related QoL (i.e., physical and psychological QoL). Participants from the EHCs tended to be more educated (*p* = 0.018), more likely to be men (*p* = 0.010) and reporting lower environmental QoL (*p* = 0.002) than their counterparts.

Due to restricted access to residential addresses and other contact details mandated by the Hong Kong Personal Data (Privacy) Ordinance [33], potential participants were approached in person and recruited upon the verification of their eligibility and provision of signed informed consent. Eligibility criteria were being a Cantonese speaker, 65+ years of age, cognitively-intact, able to walk without assistance for ≥10 m, and having lived in one of the pre-selected TPUs for at least six months. This study included 909 participants, with a response rate of 71% (1602 contacted; 322 ineligibles; 471 did not consent). Details of recruitment procedures have been described elsewhere [31].

### 2.3. Measures

#### 2.3.1. Exposures: Neighbourhood Attributes

Extant GIS data and newly-collected data from environmental audits were used to objectively assess neighbourhood environmental attributes. Extant GIS data were sourced from the Census and Statistics, Lands, and Planning Departments of the Hong Kong Special Administrative Region. To quantify relevant neighbourhood attributes using extant GIS data, participant residential buffers, representing individual neighbourhood boundaries, were created by geocoding the residential addresses of each participant and then tracing from the participants’ residences through the unique street networks in all directions for 400 and 800 m, which are considered to be walkable distances and appropriate geographical scales for older adults living in high density environments [3,23]. GIS-based environmental attributes were computed for each participant and each buffer size (i.e., 400 m and 800 m radii) using Esri’s ArcGIS software (Table S1). Given that there is no consensus about the optimal residential buffer size for studying relationships between neighbourhood attributes and older adults’ health-related outcomes [4,34], no hypotheses were formulated with regards to the buffer size that would yield stronger associations.

Environmental audits were employed to quantify neighbourhood attributes that were not assessable via extant GIS data (e.g., signs of crime/disorder) or for which the archival GIS database was outdated or incomplete. Crow-fly buffers of 400 m centred at participant residences were used as areas for environmental audits. We used 400 m crow-fly buffers rather than 400 m and 800 m street-network buffers for environmental audits for two reasons. First, study resources were insufficient to conduct environmental audits of all 400 m and 800 m street-network buffers (909 participants * 2 buffers = 1018 buffers in total) used for the computation of neighbourhood attributes based on extant GIS data. Second, previous audits conducted in Hong Kong indicated that a 400 m crow-fly distance corresponded to a street network distance from 400 to ~900 m [24,35], implying that the sizes of 400 m crow-fly residential buffers would fall between those of 400 m and 800 m street-network buffers used in this study. Environmental audits were undertaken by trained auditors using the Environment in Asian Scan Tool—Hong Kong (EAST-HK) [24] and the Public Open Space Tool (POST) [36]. To identify street segments for auditing within the 400 m crow-fly residential buffers, all segments of major roads/streets that were accessible to pedestrians were selected. If the number of selected street segments in a specific buffer was less than a quarter of the total number of segments included in that buffer, additional segments (from minor roads) were randomly selected. A validation study of the EAST-HK suggested that 25% street segments were sufficient to obtain representative estimates of various environmental attributes in Hong Kong neighbourhoods [24]. Prior to data collection, novice auditors were trained until 95% agreement between their ratings and those of experienced auditors was reached. If during data collection auditors were unsure how to rate a specific audit item, they consulted other experienced auditors in real time (i.e., shared street-segment photos and questions/comments via mobile phone) and recorded a consensual rating.

Single and multiple EAST-HK items assessed the presence of particular environmental attributes (Table S1) in each street segment defined as a section of a street between intersections. Environmental attributes were measured and aggregated by participant buffer. Scores on single-item measures denoted the percentage of audited street segments within a buffer with a particular attribute, while scores on multiple-item measures referred to the percentage of the highest obtainable score (determined by the number of items) averaged across audited street segments within a buffer. The POST assessed several features of all public parks that intersected a participant’s buffer (Table S1). These included the presence and/or location of trees, paths and amenities, and park aesthetics and visibility from surrounding areas. The maximum scores of these characteristics across all intersected parks were obtained for each participant buffer, while the total number of activity types was tallied across all parks intersecting a buffer.

#### 2.3.2. Outcome: Quality of Life

QoL was measured via interviewer-administration of the WHOQOL-BREF, the abbreviated QoL questionnaire developed by the WHO. The Hong Kong Chinese version of the WHOQOL-BREF [37] contains 26 items (24 core items and two additional items specific to Hong Kong) measuring four QoL domains: physical health (seven items), psychological health (eight items, including two Hong Kong specific items), social relationships (three items) and environment (eight items). Each item was rated on a 5-point scale. Item scores for each domain were computed and converted to standardised scores ranging from 4 to 20 according to pre-established procedures [38].

#### 2.3.3. Covariates

Covariates included in all analyses were age (years), gender (female vs. male), educational attainment (no formal education, primary school, secondary school and post-secondary school), marital status (married or cohabiting, widowed, and other), living arrangement (living with others vs. living alone), housing type (public and aided, private and renting), availability of car in the household (yes vs. no), area-level SES (high vs. low), type of recruitment centres (EHC vs. elderly community centre) and the number of diagnosed health problems. The latter was obtained using information from a clinical health-problems checklist obtained from the EHC (based on medical staff assessments) or, for those recruited at elderly community centres, from the participants at the interview.

#### 2.3.4. Analytical Approaches

Descriptive statistics were computed for all variables. Generalised additive mixed models (GAMMs) with Gaussian variance and identity link functions were used to estimate the confounder-adjusted associations between objectively-assessed physical neighbourhood attributes and QoL domains. GAMMs can model outcomes with various distributional assumptions, spatially correlated data and curvilinear relationships of unknown form [39].

First, a set of GAMMs estimated the associations of all covariates and living arrangements with QoL domain scores. Another set of main-effect GAMMs estimated the dose-response relationships of single environmental variables with QoL domain scores. Curvilinear relationships of environmental variables with the outcomes were assessed with thin-plate smoothing spline terms in GAMMs. Thin-plate splines are a type of smoothing splines that are used to model and visualize complex curvilinear relationships between continuous exposure and outcome variables. Their advantage over other smoothing spline methods is that they do not require any a priori knowledge of the functional form of the relationship of interest [39]. Smooth terms failing to provide sufficient evidence of curvilinearity, defined as a 5-unit difference in Akaike Information Criterion between a GAMM with an exposure modelled using a thin-plate spline and a GAMM with the same exposure modelled using a linear term, were replaced by simpler linear terms [39]. Moderating effects of environmental attributes on the associations between living arrangements and QoL were estimated by adding two-way interaction terms to the main-effect GAMMs. Significant interactions (*p* < 0.05) were probed using Johnson–Neyman procedures, whereby we estimated the region of significance of moderators, i.e., the range of values of the environmental attributes for which the effects of living arrangements (living alone vs. living with others) on QoL domain scores were statistically significant.

All single environmental attributes and interaction terms with *p*-value < 0.10 were entered in multiple-environmental-variable GAMMs adjusted for all covariates. We adopted a probability level of 0.10 as a criterion for the preliminary selection of environmental attributes and interaction terms to be included in multiple-environmental-variable models in order to avoid missing potentially important explanatory variables due to negative confounding. Environmental attributes that were strongly correlated were combined into composite variables as appropriate. Only those environmental attributes and interaction terms that showed a significant independent effect on the outcomes (*p* < 0.05) were retained in the final multiple-environmental-variable models. Following the recommendations of statistical theorists, no adjustments for multiple testing were performed because the analyses were confirmatory and the outcomes and environmental exposures were correlated [40,41]. All analyses were conducted in R using the packages “mgcv” and “gmodels” [42,43].

## 3. Results

Table 1 summarises the characteristics of the sample. Nearly a quarter of the sample reported living alone. Substantial levels of variability across residential buffers were observed for most of the examined environmental attributes except for signs of crime/disorder. Overall, residential buffers scored low on the presence of signs of crime/disorder and stray dogs/animals and relatively high on residential density, traffic safety, pedestrian infrastructure, presence of people, pollution and several destination-related measures.

Associations of QoL domains with socio-demographics and health conditions are shown in Table S2. Males reported higher physical and psychological QoL but lower social QoL than females. Educational attainment was positively related to all QoL domains. Widowed elders reported higher environmental QoL than those who never married or were separated/divorced. Higher environmental QoL was also reported among those having a car in their household than their counterparts. Older adults having more health problems reported lower levels of physical, psychological and social QoL.

Table 2 summarises the associations between single neighbourhood environmental attributes and QoL domains. As GIS measures of the environment based on 400 m buffers yielded weaker associations with QoL than those based on 800 m buffers, only the latter are presented in Table 2, while the former are reported in Table S3. No significant associations were found between the examined environmental attributes and physical QoL. Gross residential density was curvilinearly associated with environmental QoL only, whereby a positive association was found in those living in areas between 10,000 and 23,000 households/km^2^ (Figure S1, right top), while residents of areas <10,000 and >23,000 households/km^2^ showed nil or slightly negative associations. Entertainment density was curvilinearly associated with both psychological and environmental QoL. For psychological QoL, there was a positive association below 5 destinations/km^2^ followed by a negative association until approximately 16 destinations/km^2^. At >16 destinations/km^2^, the association was weak (large 95% confidence intervals due to a low number of observations) and showing a positive trend (Figure S1, left bottom). A similar dose-response relationship was found for environmental QoL (Figure S1, right bottom). Entertainment density within 800m buffers was also negatively, linearly associated with social QoL (Table 2). Street intersection density was negatively associated with both environmental and social QoL. The numbers of parks and activity types in parks were both negatively associated with social QoL, while the (maximum) prevalence of trees in parks was positively associated with social QoL. Density/prevalence of other types of services/destinations, connectivity, park area, pedestrian infrastructure, sitting facilities, crowdedness, presence of people and various measures of environmental aesthetics and safety were not significantly associated with any QoL domain in the whole sample.

The moderating effects of neighbourhood environmental attributes on the associations between living arrangements and QoL are summarised in Table 3, where we report the ranges of values of the environmental attributes for which the associations between living arrangements and QoL were significant at a probability level of 0.05. Compared to those living with others, older adults living alone reported lower environmental QoL when residing in neighbourhoods with poor access to non-food retail/services, food-related shops, eating outlets, health clinic/services, destinations suitable for socialising, civic/institutional destinations, fewer parks and lower levels of desirable park attributes (i.e., activity types, trees, paths and aesthetics). In contrast, when residing in neighbourhoods with more parks and activity types in parks, participants living alone reported higher environmental and social QoL than their counterparts. At high levels of greenery/natural sights (≥51.8 points), participants living alone reported lower social QoL than their counterparts. Compared to those living with others, participants living alone reported higher psychological QoL if residing in neighbourhoods with some signs of crime/disorder, while they reported lower physical QoL if living in neighbourhoods with larger park areas (≥58.8 hectares, at ~98.5th percentile). Environmental attributes that did not moderate the relationship of living arrangements with any QoL domain were: residential density, street intersection density, connectivity, entertainment density, recreation density, prevalence of public transport stops, amenities in parks, park visibility, pedestrian infrastructure, sitting facilities, crowdedness, presence of people, traffic safety, litter/decay, pollution and the presence of stray dogs/animals.

**Figure 1:**
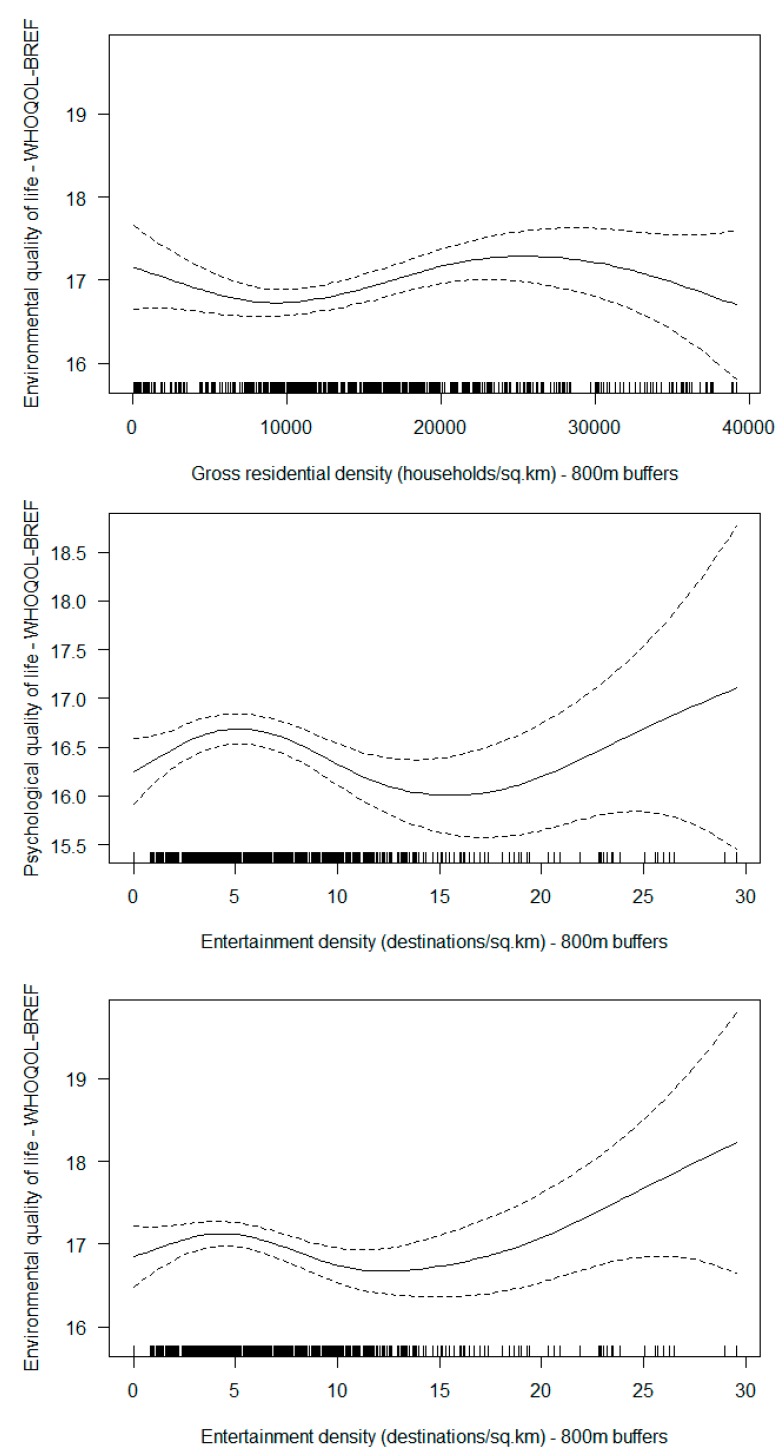
Independent curvilinear associations of neighbourhood physical attributes with quality of life (QoL). [Full lines represent point estimates of modelled scores of specific QoL dimensions, while the dotted lines represent their 95% confidence intervals.].

In multiple-neighbourhood-attribute models (Table 4), all curvilinear associations of gross residential density and entertainment density with QoL domains remained significant. The patterns of these relationships (Figure 1) resembled those of the single-neighbourhood-attribute models (Figure S1). As for linear relationships, the associations of street intersection density with environmental QoL and those of entertainment density, number of parks and park trees with social QoL remained significant. The negative association of presence of litter/decay with environmental QoL became stronger in the multiple attribute model. The neighbourhood attributes that remained significant moderators of the associations between living arrangements and QoL domains were: park area within 800 m buffers for physical QoL; signs of crime/disorder for psychological QoL; activity types in park for social QoL; and a composite destination index (a summed z-score of correlated destination attributes) for environmental QoL.

## 4. Discussion

Safe, green, aesthetically-pleasing neighbourhoods with a variety of daily destinations and services and a good-quality pedestrian infrastructure are thought to contribute to older adults’ QoL [3,21,44]. These neighbourhood characteristics are likely to be particularly important to older residents who live alone as they provide opportunities for engagement in social activities and can help them maintain their independence in activities of daily living [4,6,7,8]. Overall, this study provides partial support for these hypotheses in relation to environmental and, somewhat, social QoL, but not with respect to physical and psychological QoL.

Although very few significant associations were observed between objectively-assessed neighbourhood attributes and environmental QoL in the whole sample, most measures of destination accessibility and several measures of park quality (e.g., trees and number of park activities) modified the negative effects of living alone on environmental QoL in the expected direction. Older adults who had poor access to retail, eateries, health-related services, places for socialising, civic/institutional destinations and good-quality parks reported lower levels of environmental QoL if living alone than if living with others. These findings suggest that these neighbourhood attributes are particularly important to older residents who live alone, enabling self-reliance in performing activities of daily living. Although to our knowledge no other study has examined neighbourhood characteristics as moderators of living arrangements-QoL associations, a recent survey of Dutch elderly reported that perceived access to facilities was positively related to environmental QoL, providing further support for the importance of neighbourhood destination accessibility to older residents’ satisfaction with the environment [45].

Whilst no significant positive associations between environmental QoL and access to single destination categories were found in the whole sample, we observed a curvilinear relationship with residential density suggestive of a positive association for values ranging from ~9000 to 23,000 dwellings/km^2^ (corresponding to the samples’ 25th and 87th percentiles of density) and a nil or slightly negative association thereafter. As residential density is necessary to support local commercial and public services, it is a proxy measure of overall destination accessibility. Medium-to-high density neighbourhoods are generally characterised by mixed-use developments with a large range of complementary destinations that offer opportunities for utilitarian walking and social activities [30,46]. However, extreme levels of density are often accompanied by excessive pollution, crowding and noise [47], which may, in turn, negatively affect residents’ environmental QoL [48].

Entertainment density was the only other destination-related variable to show a significant relationship with environmental QoL in the whole sample, suggesting a positive effect for values below 5 destinations/km^2^ (40th percentile) and a negative effect for values ranging from 5 to 13 destinations/km^2^ (89th percentile) (Figure 1). A similarly-shaped relationship was also observed in relation to psychological QoL, whilst a negative linear association was found with social QoL. Neighbourhoods with several diverse entertainment destinations (e.g., a cinema, a museum and a community centre) may provide access to interesting cultural and intellectual activities that may enhance older residents’ psychological well-being and satisfaction with the environment. However, neighbourhoods with a high concentration of entertainment destinations may not provide sufficient access to other types of destination important for daily living and social QoL [6], such as affordable shops and eating outlets, which are common locations where Hong Kong older adults socialise [49]. Additionally, neighbourhoods with high entertainment density may be excessively crowded and noisy at night, tailored to younger people and their services and facilities may be overpriced for retirees. This could contribute to older adults’ feeling dissatisfied and socially isolated.

Parks and other public open spaces, where older residents can engage in physical and social activities [50], are another destination category hypothesised to benefit QoL [3,11]. However, in the fully-adjusted multiple-neighbourhood-attribute models, social QoL was negatively related to the number of parks in the neighbourhood and positively related to the prevalence of trees in parks. Additionally, the total number of activity types available in neighbourhood parks buffered the negative effects of living alone on social QoL. It appears that the mere presence of many small parks may be detrimental to social QoL because it reduces the amount of land available for other uses that promote utilitarian walking and social interactions (e.g., eateries, shops and community services). Our findings suggest that, to promote social interactions and activities among older residents, neighbourhoods should provide access to one or two well-treed parks offering opportunities for diverse recreational activities. Having parks with a mature tree canopy cover is important not only for aesthetics and restorative reasons [51,52]. A tree’s canopy acts like a parasol. It cools the surrounding environment and provides shady areas where people can meet, which are important considerations in an ultra-dense, built-up, subtropical urban environment such as Hong Kong.

It is interesting that older adults having access to a wide range of activities in local parks had higher social QoL if living alone than living with others. Parks providing different activities are likely to be more frequently visited by a larger number of residents, which increases the opportunity for older adults living alone to develop and maintain social relations with likeminded neighbours. In contrast, those living with others may commit themselves to spending more time with household members within their typically overcrowded apartment, which would result in heightened levels of stress and, thus, negative social perceptions [53,54].

Another interesting moderating effect of park accessibility on the associations between living arrangements and QoL was found in relation physical QoL. Compared to those living with others, older people living alone reported lower physical QoL when residing in neighbourhoods intersected by larger park areas. In Hong Kong, these types of neighbourhoods are located in areas adjacent to country parks or natural reserves, which are often typified by steep terrain [55] and have restricted access to public transport and daily destinations. Older adults living in such neighbourhoods have limited opportunities to engage in active travel [4] and other forms of physical activity [56], which may compromise their health and result in lower levels of physical QoL. This would be especially the case for those living alone who, in absence of support from others, need to negotiate the challenging environmental conditions they live in and may develop less favourable perceptions of their physical capacity. Yet, given that this was the only significant environmental moderating effect found with respect to physical QoL, it might have been due to chance.

Signs of crime/disorder moderated the associations between living arrangements and psychological QoL. Compared to those living with others, older people living alone reported higher psychological QoL when residing in neighbourhoods with more signs of crime/disorder. However, it should be noted that the levels of signs of crime/disorder in our study were very low (on average 0.3 out of 100 points), which is typical of Hong Kong residential neighbourhoods [57]. Some signs of crime/disorder can be observed in destination-rich, lower SES neighbourhoods [58]. Older adults living alone in these low SES areas may spend more time outside their small apartments than those living with others and capitalise on the affordable opportunities for activities offered by their communities [26], which would benefit their psychological well-being.

Two non-destination aspects of the environment that emerged as correlates of QoL domains in the whole sample were the presence of litter and/or decay and street intersection density. Whilst the former characteristic was, as anticipated, negatively associated with environmental QoL, the latter showed somewhat unexpected negative associations with environmental and social QoL. In Hong Kong, areas with a lot of street intersections (i.e., a dense street network) are also ultra-dense, filled with high-rise buildings, heavily trafficked and, hence, polluted and noisy [59], which may explain why this attribute was negatively related to older adults’ environmental QoL.

Finally, the small number of significant associations between the examined neighbourhood characteristics and QoL in the whole sample of older adults requires an explanation. Studies on factors influencing Hong Kong older adults’ physical activity, a lifestyle behaviour beneficial to QoL, identified a substantial number of neighbourhood physical attributes associated with walking within the neighbourhood [49,60] but very few attributes related to total walking [49]. Yet, it is total rather than location-specific physical activity that benefits health [61]. It has been suggested that most Hong Kong older residents living in neighbourhoods with poorer access to destinations can engage in health-enhancing levels of physical activity (and other QoL-enhancing activities) due to Hong Kong’s ubiquitous, efficient, affordable and highly integrated public transport network which allows them to get to neighbourhoods with destinations that meet their needs [49,62]. Therefore, it is possible that Hong Kong’s public transport system in conjunction with a relatively low percentage of older adults living alone might have been responsible for the overall lack of main effects observed in this study. The fact that this sample had higher average scores on the environmental and social QoL scales than those observed in other geographical locations [63] further supports for this idea, although it should be acknowledged that because the sampling strategy used in this study was not meant to provide a representative sample of the whole Hong Kong population of older adults, these QoL estimates may be biased. Pooled analyses of multi-country data with a greater variability in environmental exposures are needed to more accurately quantify dose-response relationships between neighbourhood physical attributes and QoL.

There are several strengths and limitations to our study. We assessed a large range of neighbourhood physical environmental attributes using objective methods that allowed us to control for a major potential source of reserve causality common in this research field—namely, positive affective states and better health status (high QoL) causing older adults to perceive their environment more favourably. This being a cross-sectional observational study, we cannot rule out an effect of neighbourhood self-selection bias on the findings. However, concerns about neighbourhood self-selection are mitigated by the study sampling design ensuring a balanced representation of high and low walkable neighbourhoods across SES strata [64]. Additionally, this particular threat to internal validity is alleviated by the fact that 37% of Hong Kong elderly live in public rental housing [54] and most of them have limited choice of accommodation due to Hong Kong’s sky-high residential property prices. Another study limitation pertains to the inability to employ a more comprehensive sampling frame for participant recruitment due to privacy ordinance restrictions. However, the relatively high response rate in this study alleviates concerns related to sampling bias. A further limitation is the lack of detailed information on household composition (e.g., being able to distinguish between living with a partner versus living with children or extended family) which would have helped clarify how the neighbourhood environment interacts with living conditions to affect older adults’ QoL. Further, the neighbourhood environment is likely to have stronger effects on the QoL of older adults who live alone and suffer from chronic health problems [60] than those who live alone and are relatively healthy. Therefore, it would be useful for future studies to examine the interplay of the neighbourhood environment, living arrangements and health status on older adults’ QoL. Finally, due to logistic limitations, this study used different types of residential buffers (crow-fly and street-network) to define ‘neighbourhoods’ and examined differences in associations between buffer sizes for only a small number of environmental attributes. Hence, the issue of optimal buffer size for studying environmental correlates of QoL in older adults remains unresolved. Future research in this field needs to address these limitations and aim for well-designed longitudinal and quasi-experimental studies providing more robust estimates of causal effects.

## 5. Conclusions

Medium-to-high density, well-ordered neighbourhoods with an optimal mix of well-treed public open spaces and services catering for older adults’ daily needs may significantly contribute to the social and environmental QoL of an ageing population in Hong Kong. Easy access to a variety of daily destinations and activities in local parks appears to be particularly important to the social and environmental QoL of older people living alone who are self-reliant in performing activities of daily living. The extent to which these findings are generalisable to other Asian mega-cities needs to be examined in future studies.

## Figures and Tables

**Table 1:** Sample characteristics (*N* = 909).

Variables [Theoretical Range]		Statistics (%)	
Socio-demographic and health-related characteristics			
Sex, females		66.3	
Educational attainment			
No formal education		20.8	
Primary school		35.5	
Secondary school		30.5	
Post-secondary school		13.2	
Marital status			
Married or cohabiting		59.5	
Widowed		32.7	
Other		7.8	
Housing			
Public and aided		43.1	
Private (purchased)		51.3	
Renting		5.6	
Living alone		23.1	
Household with car		28.5	
Type of recruitment centre			
Elderly health centres		82.6	
Elderly community centres		28.4	
Neighbourhood type			
Low walkable, low SES		22.0	
Low walkable, high SES		24.8	
High walkable, low SES		28.3	
High walkable, high SES		25.0	
		**Mean (SD)**	**Median (IQR) ^a^**
Age (years)		76.5 (6.0)	
Number of diagnosed health problems [0–10]		3.2 (2.0)	
*Outcome: quality of life*			
Domain 1: Physical health (score) [4–20]		16.1 (2.4)	
Domain 2: Psychological health (score) [4–20]		16.5 (2.1)	
Domain 3: Social relationships (score) [4–20]		15.1 (2.1)	
Domain 4: Environment (score) [4–20]		17.0 (2.0)	
*Environmental attributes*	Buffer		
Based on extant GIS data aggregated by street-network residential buffers			
Gross residential density (1000 households/km^2^)	400 m	15.8 (11.2)	12.2 (13.8)
	800 m	14.3 (8.4)	12.9 (11.4)
Street intersection density (intersections/km^2^)	400 m	119.9 (58.0)	
	800 m	91.5 (40.0)	
Civic and institutional density (destinations/km^2^)	400 m	88.2 (53.8)	
	800 m	69.7 (36.5)	
Entertainment density (destinations/km^2^)	400 m	11.8 (16.9)	7.3 (16.1)
	800 m	6.9 (5.2)	6.2 (6.2)
Recreation density (destinations/km^2^)	400 m	21.2 (23.2)	17.5 (30.5)
	800 m	22.5 (15.2)	20.1 (13.6)
Park area (hectares)	400 m	1.6 (9.4)	0.2 (0.9)
	800 m	9.5 (59.1)	2.0 (5.3)
Based on environmental audit data aggregated by crow-fly residential buffers			
Connectivity (score) (0–100)	400 m	40.6 (7.4)	
Prevalence of non-food retail and services (number)	400 m	15.9 (16.5)	11.0 (19.0)
Prevalence of food-related shops (number)	400 m	10.2 (8.6)	11.0 (19.0)
Prevalence of eating outlets (number)	400 m	13.6 (13.1)	9.0 (18.0)
Prevalence of destinations for socialising (number)	400 m	6.5 (6.2)	5.0 (7.0)
Prevalence of health clinics/services (number)	400 m	3.9 (4.2)	3.0 (4.0)
Prevalence of public transport stops (number)	400 m	8.1 (4.7)	7.0 (5.0)
Number of parks	400 m	2.7 (2.4)	2.0 (2.0)
Activity types in park (score)	400 m	1.8 (1.7)	2.0 (3.0)
Amenities in park (score) [0–7]	400 m	2.9 (1.4)	
Trees in park (score) [0–5]	400 m	2.1 (1.2)	
Paths in park (score) [0–6]	400 m	1.7 (1.3)	2.0 (1.0)
Park aesthetics (score) [0–3]	400 m	2.4 (1.0)	
Park visibility (score) [2–6]	400 m	2.1 (1.0)	
Pedestrian infrastructure (score) [0–100]	400 m	62.7 (9.4)	
Sitting facilities (score) [0–100]	400 m	20.5 (20.1)	17.0 (31.0)
Crowdedness (score) [0–100]	400 m	9.8 (8.8)	7.7 (12.5)
Presence of people (score) [0–100]	400 m	64.5 (21.6)	
Traffic safety (score) [0–100]	400 m	69.9 (15.0)	
Greenery/natural sights (score) [0–100]	400 m	36.9 (16.7)	45.5 (25.6)
Signs of crime/disorder (score) [0–100]	400 m	0.3 (0.9)	0.0 (0.0)
Stray dogs/animals (score) [0–100]	400 m	5.9 (9.9)	0.0 (9.0)
Litter/decay (score) [0–100]	400 m	22.9 (4.1)	
Pollution (score) [0–100]	400 m	42.3 (33.2)	40.0 (61.2)
Number of street segments audited	400 m	21.4 (17.5)	16.0 (13.0)

SES = socio-economic status; SD = standard deviation; IQR = interquartile range; GIS geographic information systems; ^a^ computed for environmental variables with skewness > |1.0|.

**Table 2:** Associations of single neighbourhood physical environmental attributes with quality of life (QoL) domains.

Environmental Attributes (Unit)	Physical QoL		Psychological QoL		Social QoL		Environmental QoL	
	*b* (95% *CI*)	*p*	*b* (95% *CI*)	*p*	*b* (95% *CI*)	*p*	*b* (95% *CI*)	*p*
Gross residential density ^a^ (1000 households/km^2^)	−0.007 (−0.028, 0.013)	0.485	0.011 (−0.006, 0.028)	0.217	−0.001 (−0.018, 0.016)	0.914	−0.696 (−1.419, 0.027)	0.059
- Curvilinear ^†^	-	-	-	-	-	-	*F*_(3.318, 3.318)_ = 2.847 *	0.029
Street intersection density ^a^ (100 intersections/km^2^)	−0.138 (−0.576, 0.300)	0.537	−0.325 (−0.685, 0.034)	0.076	−0.396 (−0.744, −0.047) *	0.026	−0.731 (−1.083, −0.379) ***	<0.001
Connectivity ^b^ (score)	−0.001 (−0.027, 0.025)	0.945	−0.001 (−0.022, 0.021)	0.962	0.005 (−0.016, 0.026)	0.646	0.003 (−0.018, 0.024)	0.781
Civic and institutional density ^a^ (destinations/km^2^)	0.001(−0.004, 0.006)	0.663	0.000 (−0.003, 0.004)	0.818	−0.002 (−0.006, 0.002)	0.258	−0.003 (−0.006, 0.001)	0.206
Prevalence of non-food retail/services ^b^ (number)	−0.004 (−0.016, 0.009)	0.572	−0.008 (−0.018, 0.002)	0.130	−0.008 (−0.018, 0.003)	0.143	−0.005 (−0.015, 0.005)	0.332
Entertainment density ^a^ (destinations/km^2^)	−0.015 (−0.047, 0.017)	0.369	0.512 (−0.277, 1.301)	0.203	−0.042 (−0.068, -0.016) **	0.002	0.528 (−0.248, 1.30)	0.182
- Curvilinear ^†^	-	-	*F*_(3.572, 3.572)_ = 3.332 **	0.009	-	-	*F*_(3.652, 3.652)_ = 3.706 **	0.004
Recreation density ^a^ (destination/km^2^)	−0.008 (−0.019, 0.003)	0.152	−0.002 (−0.011, 0.007)	0.679	−0.001 (−0.010, 0.008)	0.861	0.000 (−0.009, 0.009)	0.988
Prevalence of food-related shops ^b^ (number)	−0.015 (−0.040, 0.011)	0.257	−0.013 (−0.033, 0.007)	0.198	−0.016 (−0.036, 0.003)	0.100	0.001 (−0.020, 0.022)	0.929
Prevalence of eating outlets ^b^ (number)	−0.000 (−0.019, 0.018)	0.971	−0.004 (−0.019, 0.011)	0.626	−0.002 (−0.017, 0.013)	0.774	0.001 (−0.014, 0.016)	0.928
Prevalence of destinations for socialising ^b^ (number)	−0.012 (−0.045, 0.022)	0.494	-0.010 (−0.037, 0.018)	0.497	−0.016 (−0.042, 0.011)	0.243	−0.005 (−0.032, 0.023)	0.737
Prevalence of health clinics/services ^b^ (number)	−0.006 (−0.052, 0.039)	0.788	−0.018 (−0.056, 0.019)	0.335	−0.029 (−0.066, 0.007)	0.111	−0.000 (−0.037, 0.037)	0.996
Prevalence of public transport stops ^b^ (number)	−0.024 (−0.074, 0.026)	0.355	−0.002 (−0.043, 0.039)	0.927	−0.026 (−0.065, 0.013)	0.194	0.003 (−0.037, 0.044)	0.875
Parks ^b^ (number)	−0.031 (−0.100, 0.038)	0.383	−0.045 (−0.101, 0.012)	0.123	−0.080 (−0.135, −0.025) **	0.005	−0.026 (−0.085, 0.034)	0.398
Park area ^a^ (hectares)	−0.000 (−0.003, 0.002)	0.852	0.000 (−0.002, 0.002)	0.998	−0.002 (−0.004, −0.000)	0.112	0.000 (−0.002, 0.003)	0.776
Activity types in park ^b^ (number)	−0.031 (−0.127, 0.066)	0.537	−0.018 (−0.098, 0.062)	0.657	−0.096 (−0.175, −0.018) *	0.016	−0.044 (−0.127, 0.039)	0.296
Amenities in park ^b^ (score)	−0.049 (−0.175, 0.077)	0.448	0.006 (−0.100, 0.112)	0.911	0.043 (−0.060, 0.147)	0.414	−0.006 (−0.114, 0.103)	0.918
Trees in park ^b^ (score)	0.074 (−0.085, 0.233)	0.359	0.053 (−0.080, 0.186)	0.439	0.132 (0.003, 0.261) *	0.046	−0.018 (−0.154, 0.118)	0.799
Paths in park ^b^ (score)	0.072 (−0.083, 0.226)	0.362	0.062 (−0.067, 0.192)	0.347	0.046 (−0.080, 0.173)	0.470	0.006 (−0.126, 0.137)	0.934
Park aesthetics ^b^ (score)	−0.031 (−0.210, 0.148)	0.734	−0.003 (−0.154, 0.147)	0.964	0.087 (−0.060, 0.234)	0.243	0.052 (−0.102, 0.205)	0.509
Park visibility ^b^ (score)	−0.022 (−0.217, 0.173)	0.823	−0.016 (−0.178, 0.145)	0.843	0.131 (−0.026, 0.288)	0.102	0.022 (−0.145, 0.190)	0.792
Pedestrian infrastructure ^b^ (score)	0.008 (−0.013, 0.028)	0.462	0.001 (−0.015, 0.016)	0.938	0.004 (−0.012, 0.019)	0.647	0.010 (−0.006, 0.026)	0.220
Sitting facilities ^b^ (score)	−0.004 (−0.015, 0.006)	0.418	−0.003 (−0.011, 0.005)	0.397	0.001 (−0.007, 0.009)	0.770	−0.002 (−0.010, 0.006)	0.614
Crowdedness ^b^ (score)	−0.016 (−0.036, 0.005)	0.131	−0.010 (−0.027, 0.006)	0.218	−0.010 (−0.026, 0.006)	0.228	−0.005 (−0.021, 0.012)	0.567
Presence of people ^b^ (score)	−0.005 (−0.014, 0.004)	0.248	0.000 (−0.007, 0.008)	0.927	−0.003 (−0.011, 0.004)	0.394	0.000 (−0.007, 0.008)	0.915
Traffic safety ^b^ (score)	0.000 (−0.012, 0.013)	0.968	0.002 (−0.008, 0.013)	0.655	−0.002 (−0.013, 0.008)	0.628	0.004 (−0.007, 0.014)	0.484
Greenery/natural sights ^b^ (score)	−0.005 (−0.023, 0.013)	0.565	−0.007 (−0.022, 0.008)	0.377	−0.001 (−0.015, 0.014)	0.909	0.004 (−0.011, 0.019)	0.079
Signs of crime/disorder ^b^ (score)	−0.023 (−0.221, 0.176)	0.823	−0.084 (−0.246, 0.077)	0.306	−0.080 (−0.238, 0.078)	0.322	−0.020 (−0.183, 0.143)	0.808
Stray dogs/animals ^b^ (score)	−0.009 (−0.028, 0.009)	0.320	−0.008 (−0.023, 0.007)	0.291	−0.000 (−0.015, 0.014)	0.992	−0.002 (−0.017, 0.013)	0.794
Litter/decay ^b^ (score)	−0.011 (−0.056, 0.034)	0.632	−0.035 (−0.070, 0.000)	0.053	−0.031 (−0.065, 0.004)	0.080	−0.035 (−0.071, 0.001)	0.059
Pollution ^b^ (score)	−0.000 (−0.006, 0.005)	0.902	−0.002(−0.006, 0.003)	0.459	−0.003 (−0.008, 0.001)	0.112	−0.003 (−0.007, 0.002)	0.234

*b* = regression coefficient; CI = confidence interval; *p* = *p*-value; - = not applicable. ^a^ based on extant geographic information systems data—measure computed using 800 m street-network residential buffers; ^b^ based on data from environmental audits—measure computed using 400 m crow-fly residential buffers; ^†^ curvilinear associations depicted in Figure S1 (Additional file 1). All estimates adjusted for age, sex, educational attainment, household with car, marital status, housing type, living arrangement, area-level socio-economic status, type of recruitment centre, and number of current diagnosed health problems. “0.000” occurs due to rounding and does not equal to zero. * *p* < 0.05; ** *p* < 0.01; *** *p* < 0.001.

**Table 3:** Associations between living arrangements (reference group: living with others) and quality of life (QoL) domains at region-of-significance threshold values of neighbourhood physical environmental attributes (moderators).

Environmental Attribute	QoL Domain	Region-of-Significance Value of Environmental Moderator [% SRSV]	*b* (95% *CI*)
Civic and institutional density ^a^	Environmental	≤44.0 locations/km^2^ [23%]	−0.405 (−0.809, −0.000) *
Prevalence of non-food retail/services ^b^	Environmental	≤1.3 destinations/bf [14%]	−0.446 (−0.892, −0.000) *
Prevalence of food-related shops ^b^	Environmental	≤4.0 shops/bf [35%]	−0.441 (−0.821, −0.002) *
Prevalence of eating outlets ^b^	Environmental	≤1.4 outlets/bf [14%]	−0.443 (−0.884, −0.001) *
Prevalence of destinations for socialising ^b^	Environmental	≤0.8 destinations/bf [9%]	−0.446 (−0.892, 0.000) *
Prevalence of health clinics/services ^b^	Environmental	≤1.6 destinations/bf [35%]	−0.388 (−0.773, −0.004) *
		≥11.6 destinations/bf [8%]	0.693 (0.004, 1.383) *
Number of parks ^b^	Social	≤0.9 parks/bf [13%]	−0.429 (−0.850, −0.008) *
		≥11.7 parks/bf [2%]	1.176 (0.002, 2.351) *
	Environmental	≤1.3 parks/bf [25%]	−0.390 (−0.773, −0.006) *
		≥10.2 parks/bf [2%]	0.962 (0.000, 1.923) *
Park area ^a^	Physical	≥58.8 hectares [2%]	−0.913 (−1.825, −0.000) *
Activity types in park ^b^	Psychological ^†^	Minimum: 0 types [69%]	−0.392 (−0.880, 0.097) ^#^
		Maximum: 9 types [2%]	1.289 (−0.101, 2.678) ^‡^
	Social	≤0.8 types/bf [31%]	−0.412 (−0.810, −0.013) *
		≥5.6 types/bf [3%]	0.785 (0.001, 1.568) *
	Environmental	≤0.8 types/bf [31%]	−0.388 (−0.771, −0.006) *
		≥7.3 types/bf [2%]	1.026 (0.005, 2.046) *
Trees in park ^b^	Environmental	≤1.3 points [18%]	−0.419 (−0.822, −0.016) *
Paths in park ^b^	Environmental	≤0.7 points [14%]	−0.427 (−0.845, −0.008) *
Park aesthetics ^b^	Environmental	≤1.9 points [13%]	−0.427 (−0.834, -0.019) *
Greenery/natural sights ^b^	Social	≥51.8 points [3%]	−0.454 (−0.908, −0.000) *
Signs of crime/disorder ^b^	Psychological	≥2.0 points [7%]	0.752 (0.013, 1.492) *

*Notes. b* = regression coefficient; *CI* = confidence interval; % SRS = % of sample falling within the region of significance values. ^a^ based on extant geographic information systems data—measure computed using 800 m street-network residential buffers; ^b^ based on data from environmental audits—measure computed using 400 m crow-fly residential buffer. bf = buffer. † Given no region-of-significance found, estimates at minimum and maximum values of moderator are shown. All estimates adjusted for age, gender, educational attainment, household with car, marital status, housing type, area-level socio-economic status, type of recruitment centre, and number of current diagnosed health problems. “0.000” occurs due to rounding and does not equal to zero. Only significant (*p* < 0.05) interaction terms between living arrangements and specific neighbourhood environmental attributes are shown. * *p* = 0.050; ^#^ *p* = 0.116; ^‡^ *p* = 0.069.

**Table 4:** Associations of multiple neighbourhood physical environmental attributes with quality of life (QoL) domains.

Variables ^†^	Physical QoL		Psychological QoL		Social QoL		Environmental QoL	
	*b* (95% *CI*)	*p*	*b* (95% *CI*)	*p*	*b* (95% *CI*)	*p*	*b* (95% *CI*)	*p*
ENVIRONMENTAL MAIN EFFECTS								
*Variable not interacting with living arrangement (measurement unit)*								
Gross residential density ^a^ (1000 households/km^2^)—curvilinear	-	-	-	-	-	-	*F*_(3.281, 3.281)_ = 3.342 *	0.018
Street intersection density ^a^ (100 intersections/km^2^)	-	-	-	-	-	-	−0.640 (−1.077, −0.204) **	0.004
Entertainment density ^a^ (destinations/km^2^)	-	-	-	-	−0.036 (−0.062, −0.009) **	0.009	-	-
Entertainment density ^a^ (destinations/km^2^)—curvilinear	-	-	*F*_(3.582, 3.582)_ = 3.017 *	0.014	-	-	*F*_(3.525, 3.525)_ = 2.264 *	0.039
Number of parks ^b^	-	-	-	-	−0.097 (−0.186, −0.008) *	0.032	-	-
Trees in park ^b^ (score)	-	-	-	-	0.160 (0.030, 0.289) *	0.016	-	-
Litter/decay ^b^ (score)	-	-	-	-	-	-	−0.035 (−0.068, −0.002) *	0.038
*Variable interacting with living arrangement*								
Composite destination index ^‡^	-	-	-	-	-	-	−0.016 (−0.056, 0.023)	0.409
Park area ^a^ (hectares)	0.000 (−0.002, 0.003)	0.813	-	-	-	-	-	-
Activity types in park ^b^ (number)	-	-	-	-	−0.082 (−0.205, 0.040)	0.188	-	-
Signs of crime/disorder ^b^ (score)	-	-	−0.187 (−0.371, −0.004) *	0.045	-	-	-	-
LIVING ARRANGEMENTS BY ENVIRONMENT INTERACTION EFFECTS								
*Effect of living arrangements (reference group: living with others) at region-of-significance threshold values of environmental attributes* [% SRSV]								
Composite destination index ^‡^								
0.01 level: ≤−6.8 z-scores [4%]	-	-	-	-	-	-	−0.679 (−1.193, −0.164) **	-
0.05 level: ≤−3.3 z-scores [33%]	-	-	-	-	-	-	−0.393 (−0.782, −0.004) *	-
0.05 level: ≥9.4 z-scores [6%]	-	-	-	-	-	-	0.644 (0.003, 1.285) *	-
Park area ^a^								
0.05 level: ≥58.8 hectares [2%]	−0.913 (−1.825, −0.000) *	-	-	-	-	-	-	-
Activity types in park ^b^								
0.05 level: ≤0.8 types [31%]	-	-	-	-	−0.409 (−0.805, −0.014) *	-	-	-
0.05 level: ≥6.2 types [2%]	-	-	-	-	0.883 (0.003, 1.762) *	-	-	-
Signs of crime/disorder ^b^								
0.05 level: ≥2.2 points [5%]	-	-	0.815 (0.010, 1.620) *	-	-	-	-	*-*

*Notes. b* = regression coefficient; *CI* = confidence interval; *p* = *p*-value; .05 or .01 level = significance levels; - = not applicable; % SRS = % of sample falling within the region of significance values. ^a^ based on extant geographic information systems data—measure computed using 800 m street-network residential buffers; ^b^ based on data from environmental audits—measure computed using 400 m crow-fly residential buffer. ^†^ associations were linear unless specified, and curvilinear associations are depicted in Figure 1. ^‡^ the sum of z-scores of single destination-related variables that interacted with living arrangement in the single-environmental-variable models, including civic and institutional density—800 m street-network buffer, prevalence of non-food retail/services, prevalence of food-related shops, prevalence of eating outlets, prevalence of destinations for socialising, and prevalence of health clinics/services. All estimates adjusted for age, gender, educational attainment, household with car, marital status, housing type, area-level socio-economic status, type of recruitment centre, number of current diagnosed health problems, and other environmental attributes that uniquely contributed to the explanation of the outcome variables through main or moderating effects. “0.000” occurs due to rounding and does not equal to zero. * *p* < 0.05; ** *p* < 0.01.

## Supplementary Materials

The following are available online at https://www.mdpi.com/1660-4601/16/5/876/s1, Figure S1: Curvilinear associations of single neighbourhood attributes with quality of life (QoL), Table S1: Definitions of environmental variables and expected associations with quality of life (QoL), Table S2: Associations of socio-demographic and health-related characteristics with quality of life (QoL) domains, Table S3: Associations between single GIS neighbourhood environmental attributes based on 400 m street-network buffers and quality of life (QoL) domains.
