# Supplementary material for: Objectively-Measured Neighbourhood Attributes as Correlates and Moderators of Quality of Life in Older Adults with Different Living Arrangements: The ALECS Cross-Sectional Study
Source: Int J Environ Res Public Health. 2019 Mar 10;16(5):876. doi: 10.3390/ijerph16050876 (PMC6427272; doi:10.3390/ijerph16050876)
Supplement: Supplementary file 1 [file ijerph-16-00876-s001.pdf]

**Supplementary Materials**

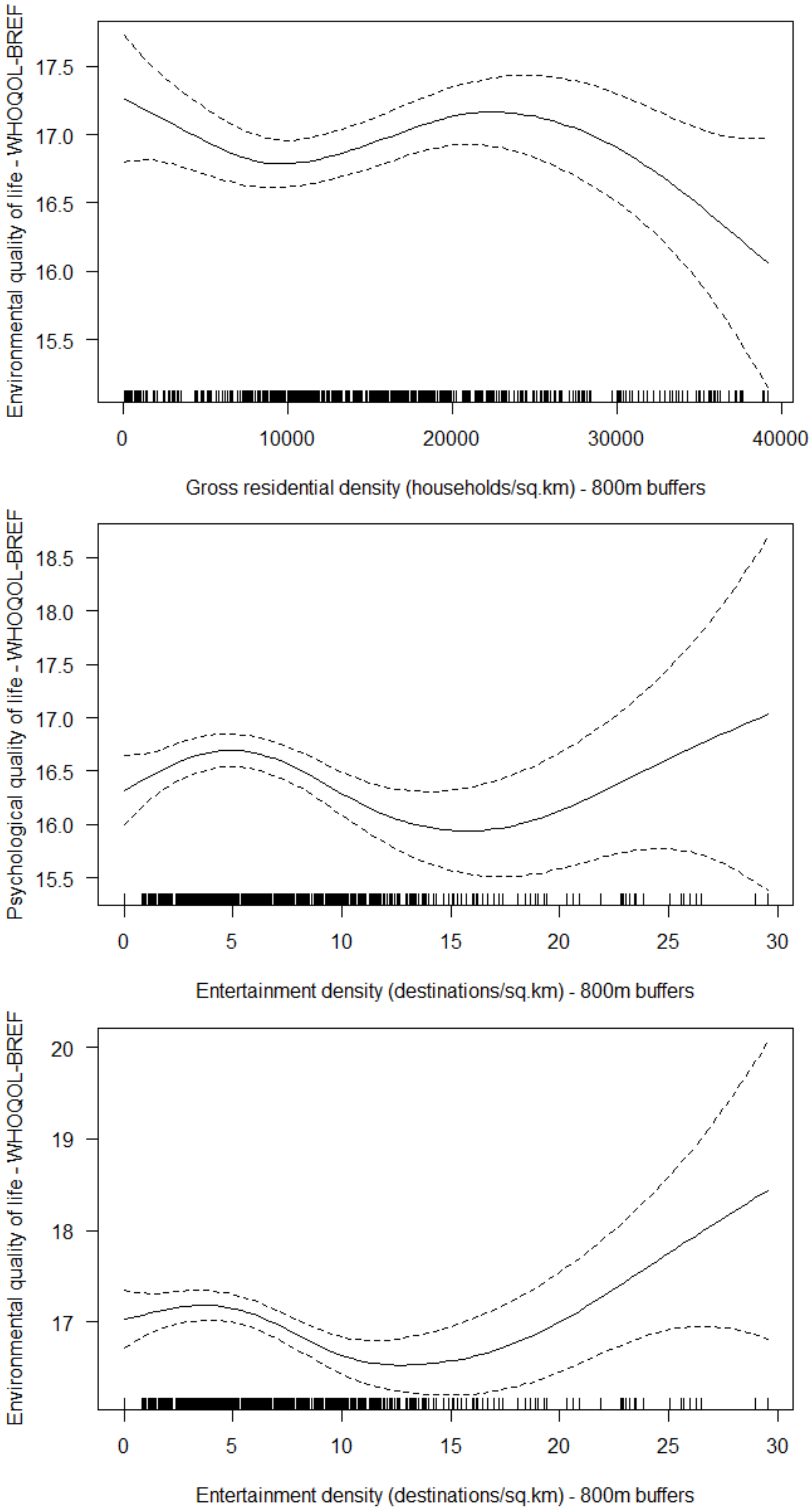


**Figure S1.** Curvilinear associations of single neighbourhood attributes with quality of life (QoL)—right top: association between gross residential density and environmental QoL; left bottom: association between entertainment density and psychological QoL; right bottom: association between entertainment density and environmental QoL. Notes: the solid lines represent point estimates (and dashed lines represent their 95% confidence intervals) of WHOQOL−BREF scores at various values of neighbourhood attributes.

**Table S1.** Definitions of environmental variables and expected associations with quality of life (QoL).

| Environmental Variable (Data Source; Type of Buffer Used) | Definition | Expected Associations with Quality of Life |
|---|---|---|
| Participant street-network residential buffer (extant GIS) | An irregularly shaped polygon around a participant's home address (geocoded) that approximates neighbourhood boundaries. Buffer polygons were created for two distances (400-m and 800-m) by tracing through unique street networks in all directions. We calculated the total land area (km$^2$) of each participant residential buffer. All GIS variables listed below were computed for each participant's 400 m and 800 m street-network buffers. | Not applicable |
| Gross residential density (extant GIS; 400 m and 800 m street-network buffers) | Number of residential households divided by the area of the participant street−network residential buffers, expressed as households per km$^2$. | Positive or rotated J-shaped<br>Providing better access to destinations, more opportunities for physical activity [1] and social interactions [2] that are associated with better perceived QoL [3]. However, very high levels of density may act as stressors [4] which have a negative effect on QoL [5]. |
| Street intersection density (extant GIS; 400 m and 800 m street-network buffers) | Number of three−and−more−way intersections divided by the area of the participant street−network residential buffers, expressed as intersections per km$^2$. | Positive or rotated J-shaped<br>Providing better access to destinations, more opportunities for physical activity and social interactions that are associated with better perceived QoL. However, moderate-to-high levels of these attributes may be associated with higher traffic-related hazards and noise [6] which have been found to be negatively related to QoL [7]. |
| Connectivity (EA; 400 m crow-fly buffer) | Assessed by 2 items: no cul-de-sacs; bridge/overpass or tunnel. A participant's score on this variable represents the percentage of a street−segment's highest obtainable score (in this case, 2) averaged across audited street segments within a participant crow−fly residential buffer. | |
| Civic and institutional density (extant GIS; 400 m and 800 m street-network buffers) | Number of civic and institutional locations (e.g., government office, public services, school) divided by the area of the participant street-network residential buffers, expressed as number of destinations per km$^2$. | Positive<br>Access to civic/institutional destinations provides more opportunities for active transport [6] and social activities that are associated with better QoL [3,8]. |
| Prevalence of non-food retail and services (EA; 400m crow-fly buffer) | Sum of number of non-food retail and services types (post-office, hotel, warehouse, laundry, bank, hardware store, pharmacy, clothing/shoe store, book/stationary store and video/audio store) across all audited street segments within a participant's crow-fly residential buffer. | Positive<br>Access to retail/commercial destinations provides more opportunities for active transport and social activities. |
| Entertainment density (extant GIS; 400 m and 800m street-network buffers) | Number of entertainment destinations (e.g., theatre, museum, community centre, art gallery) divided by the area of the participant street−network residential buffers, expressed as number of destinations per km$^2$. | Positive<br>Access to entertainment destinations provides more opportunities for active transport and social and leisure activities that are associated with better perceived QoL [3,8,9]. |

| | | |
|---|---|---|
| Recreation density (extant GIS; 400 m and 800 m street-network buffers) | Number of recreational destinations (e.g., sports centre, swimming pool, fitness club) divided by the area of the participant street-network residential buffers, expressed as number of destinations per km$^2$. | Positive<br>Access to recreational destinations provides more opportunities for active transport, leisure-time physical activity and social activities. |
| Prevalence of food-related shops (EA; 400 m crow-fly buffer) | Sum of number of food-related shop types (convenience store, supermarket, fresh-food market and bakery/cake shop) across all audited street segments within a participant's crow-fly residential buffer. | Positive<br>Access to food-related destinations provides more opportunities for active transport and social activities. |
| Prevalence of eating outlets (EA; 400 m crow-fly buffer) | Sum of number of eating outlet types (chained fast−food restaurant, Chinese coffee/noodle shop, Chinese non-fast-food restaurant, Western non-fast-food restaurant and Western coffee shop) across all audited street segments within a participant's crow-fly residential buffer. | |
| Prevalence of destinations for socialising (EA; 400 m crow-fly buffer) | Sum of number of destinations suitable for socialising (community/elderly centre, museum, HK Jockey Club betting branch, movie or theatre, hairdresser or barber, religious places and library) across all audited street segments within a participant's crow-fly residential buffer. | Positive<br>Access to destinations where older adults can socialise and meet others provides more opportunities for active transport and social activities. |
| Prevalence of health clinics/services (EA; 400m crow-fly buffer) | Total number of health clinics/services in all audited street segments within a participant's crow-fly residential buffer. | Positive<br>Access to health−related destinations provides more opportunities for active transport and social activities. |
| Prevalence of public transport stops (EA; 400 m crow-fly buffer) | Total number of public transport stops (bus stop, tram stop, MTR/train stop, ferry) in all audited street segments within a participant's crow-fly residential buffer. | Positive or rotated J-shaped<br>Providing better access to destinations, more opportunities for active transport and social interactions. However, high levels of public transport density may be associated with higher traffic-related noise and pollution and these have negative effects on QoL [9]. |
| Number of parks (EA; 400 m crow-fly buffer) | Number of public parks intersecting a participant's crow-fly residential buffer. A public park was defined as a government designed park of any size that was free of charge, open to the public and maintained by a governmental agency. | Positive<br>Access to parks provides more opportunities for active transport, leisure-time physical activity and social and leisure activities. |
| Park area (extant GIS; 400 m and 800 m street-network buffers) | Total area (hectare) of public parks intersecting the participant street−network residential buffers. | Positive<br>Access to open space and good quality of parks provide more opportunities for active transport, leisure-time physical activity and social and leisure activities. |
| Activity types in park (EA; 400 m crow-fly buffer) | Total number of activity types across all public parks intersecting a paritcipant's crow−fly residential buffer. | |
| Amenities in park (EA; 400m crow-fly buffer) | Maximum number of amenities across all public parks intersecting a participant's crow-fly residential buffer. Amenities are assessed by 7 items: chidren's play equipment, seating facilities, dog litter bags, taps/water sources | |

| | | |
|---|---|---|
| | for dogs, drinking fountains, parking facilities, and public transport. The maximum possible score is 7. | |
| Trees in park (EA; 400 m crow-fly buffer) | Maximum score of tree scale across all public parks intersecting a participant's crow−fly residential buffer. Tree scale assesses the tree placement at all perimeter sides and within parks, with higher scores indicating wider tree pattern. The maximum possible score is 5. | |
| Paths in park (EA; 400 m crow-fly buffer) | Maximum score of path scale all across public parks intersecting a participant's crow-fly residential buffer. Path scale assesses the path placement in terms of perimeter sides and diagonal or radial shapes across a park, with higher scores indicating wider path pattern. The maximum possible score is 7. | |
| Park aesthetics (EA; 400 m crow-fly buffer) | Maximum score of aesthetics across all public parks intersecting a participant's crow−fly residential buffer. Aesthetics is assessed by 3 items: watered grass, no graffiti, and no vandalism. The maximum possible score is 3. | |
| Park visibility (EA; parks within 400 m crow-fly buffer) | Maximum score of visibility across all public parks intersecting a participant's crow-fly residential buffer. Visibiltiy from both surrounding roads and surrounding buildings/houses are assessed. Three levels are used: clearly seen, partly seen, and cannot be seen. The maximum possible score is 8. | |
| Pedestrian infrastructure (EA; 400 m crow-fly buffer) | Assessed by 7 items: no steep roads/hilly street, footpaths present, footpaths well-maintained, no wet and slippery streets, bridge/overpass or tunnel, no major barriers to walking, and presence of indoor air-conditioned areas for walking. A participant's score on this variable represents the percentage of a street-segment's highest obtainable score (in this case, 7) averaged across audited street segments within a participant crow-fly residential buffer. | Positive<br>Providing better access to destinations, more opportunities for active transport and social interactions. |
| Sitting facilities (EA; 400 m crow−fly buffer) | Assessed by a single item: benches/places for sitting. This variable represents the percentage of street-segments within a participant crow-fly residential buffer that have sitting facilities. | Positive<br>Providing a place to rest outdoors and socialise with others. |
| Crowdedness (EA; 400 m crow-fly buffer) | Assessed by 3 items: street crowded, motor vehicles parked on the footpaths, and hawkers and shops on streets. A participant's score on this variable represents the percentage of a street-segment's highest obtainable score (in this case, 3) averaged across audited street segments within a participant crow-fly residential buffer. | Positive or rotated J−shaped<br>Providing opportunities for active transport and social interactions. However, high levels of crowdedness may be associated with higher levels of noise and stress. |
| Presence of people (EA; 400 m crow-fly buffer) | Assessed by 4 items: presence of adults or teenagers, elders, children, and people talking and greeting each other. A participant's score on this variable represents the percentage of a street-segment's highest obtainable score (in this case, 4) averaged across audited street segments within a participant crow-fly residential buffer. | Positive<br>Providing opportunities for social interactions. |

| | | |
|---|---|---|
| Traffic safety (EA; 400 m crow-fly buffer) | Assessed by 5 items: dirt/grass strip separating traffic from footpath, no aggressive drivers, street crossing aids, no parked cars blocking view of incoming traffic, and traffic calming devices (stop light, traffic island, crosswalk). A participant's score on this variable represents the percentage of a street-segment's highest obtainable score (in this case, 5) averaged across audited street segments within a participant crow-fly residential buffer. | Positive<br>Providing a safe environment for walking for transport and recreation. |
| Greenery/natural sights (EA; 400 m crow-fly buffer) | Assessed by 2 items: trees along street segment, and attractive natural sights. A participant's score on this variable represents the percentage of a street-segment's highest obtainable score (in this case, 2) averaged across audited street segments within a participant crow-fly residential buffer. | Positive<br>Exposure to greenery and aesthetically-pleasing sceneries may improve life satisfaction [10] by promoting engagement of physical and social activities. |
| Signs of crime/disorder (EA; 400 m crow-fly buffer) | Assessed by 5 items: people fighting, homeless people, prostitutes, needles/syringes, and graffiti. A participant's score on this variable represents the percentage of a street-segment's highest obtainable score (in this case, 5) averaged across audited street segments within a participant crow-fly residential buffer. | Negative<br>Increase feelings of threat and act as deterrents to walking and spending time outdoors. |
| Stray dogs/animals (EA; 400 m crow-fly buffer) | Assessed by a single item: presence of stray dogs/animals. This variable represents the percentage of street-segments within a participant crow-fly residential buffer where stray dogs/animals were observed. | Negative<br>Increase feelings of threat and act as deterrents to walking and spending time outdoors. |
| Litter/decay (EA; 400 m crow-fly buffer) | Assessed by 5 items: litter, broken bottles and cans, dog/animal fouling, no attractive buildings, and abandoned/vacant buildings. A participant's score on this variable represents the percentage of a street-segment's highest obtainable score (in this case, 5) averaged across audited street segments within a participant crow−fly residential buffer. | Negative<br>Increase feelings of threat and act as deterrents to walking and spending time outdoors. |
| Pollution (EA; 400 m crow-fly buffer) | Assessed by 2 items: noise pollution, and unpleasant odour. A participant's score on this variable represents the percentage of a street-segment's highest obtainable score (in this case, 2) averaged across audited street segments within a participant crow-fly residential buffer. | Negative<br>Acts as a deterrent to walking and spending time outdoors and decrease perceived QoL [7]. |

*Notes:* GIS = geographic information systems; EA = environmental audits.

## References


1. Sallis JF, Cerin E, Conway TL, et al. Physical activity in relation to urban environments in 14 cities worldwide: A cross−sectional study. *Lancet* 2016, 387, 2207−17.
2. Zhu X, Yu CY, Lee C, et al. A retrospective study on changes in residents' physical activities, social interactions, and neighborhood cohesion after moving to a walkable community. *Prev. Med*. 2014, 69 Suppl 1, S93−7.
3. Gouveia ERQ, Gouveia BR, Ihle A, et al. Correlates of health−related quality of life in young−old and old−old community−dwelling older adults. *Qual Life Res*. 2017, 26, 1561−9.
4. Fleming I, Baum A, Weiss L. Social density and perceived control as mediators of crowding stress in high−density residential neighborhoods. *J Pers Soc Psychol*. 1987, 52, 899−906.

5. Almeida DM, Piazza JR, Stawski RS, et al. The speedometer of life: Stress, health and aging. In: Schaie KW, Willis SL, editors. Handbook of the psychology of aging. 7 ed. San Diego, CA: Academic Press; 2011. p. 191−206.
6. Cerin E, Nathan A, van Cauwenberg J, et al. The neighbourhood physical environment and active travel in older adults: a systematic review and meta−analysis. *Int J Behav Nutr Phys Act.* 2017, 14, 15.
7. Oiamo TH, Luginaah IN, Baxter J. Cumulative effects of noise and odour annoyances on environmental and health related quality of life. Soc Sci Med. 2015, 146, 191−203.
8. Van Cauwenberg J, Van Holle V, De Bourdeaudhuij I, et al. Neighborhood walkability and health outcomes among older adults: The mediating role of physical activity. *Health Place.* 2016, 37, 16−25.
9. Kim J, Irwin L, Kim M, et al. The Role of Leisure Engagement for Health Benefits Among Korean Older Women. *Health Care Women Int*. 2015, 36, 1357−74.
10. De Vries S, Van Dillen SM, Groenewegen PP, et al. Streetscape greenery and health: stress, social cohesion and physical activity as mediators. *Soc Sci Med*. 2013, 94, 26−33.


**Table S2.** Associations of socio−demographic and health−related characteristics with quality of life (QoL) domains.

| Characteristics | Physical QoL | | Psychological QoL | | Social QoL | | Environmental QoL | |
|---|---|---|---|---|---|---|---|---|
| | ***b* (95% *CI*)** | ***p*** | ***b* (95% *CI*)** | ***p*** | ***b* (95% *CI*)** | ***p*** | ***b* (95% *CI*)** | ***p*** |
| Age | −0.010 (−0.038, 0.019) | 516 | −0.004 (−0.030, 0.022) | 0.741 | 0.002 (−0.023, 0.028) | 0.853 | 0.007 (−0.018, 0.031) | 0.591 |
| Sex | | | | | | | | |
| Female † | – | – | – | – | – | – | – | – |
| Male | 0.653 (0.293, 1.014) *** | <0.001 | 0.378 (0.051, 0.704) * | 0.023 | −0.471 (−0.790, −0.152) ** | 0.004 | 0.252 (−0.054, 0.558) | 0.106 |
| Education attainment | | | | | | | | |
| No formal education † | – | – | – | – | – | – | – | – |
| Primary school | 0.450 (0.007, 0.893) * | 0.046 | 0.189 (−0.212, 0.589) | 0.356 | 0.127 (−0.265, 0.518) | 0.526 | 0.425 (0.050, 0.802) * | 0.026 |
| Secondary school | 0.680 (0.202, 1.159) ** | 0.005 | 0.433 (0.001, 0.865) * | 0.050 | 0.483 (0.061, 0.906) * | 0.025 | 0.636 (0.230, 1.042) ** | 0.002 |
| Post-secondary school | 0.877 (0.287, 1.466) ** | 0.004 | 0.781 (0.250, 1.311) ** | 0.004 | 0.860 (0.341, 1.379) ** | 0.001 | 0.963 (0.462, 1.464) *** | <0.001 |
| Marital status | | | | | | | | |
| Married or cohabiting † | – | – | – | – | – | – | | |
| Widowed | 0.081 (−0.315, 0.477) | 0.687 | −0.103 (−0.461, 0.255) | 0.571 | −0.128 (−0.478, 0.221) | 0.471 | 0.160 (−0.176, 0.496) | 0.350 |
| Other | 0.184 (−0.421, 0.790) | 0.551 | −0.188 (−0.737, 0.360) | 0.500 | −0.179 (−0.715, 0.357) | 0.512 | −0.434 (−0.948, 0.080) | 0.098 |
| Housing | | | | | | | | |
| Public and aided † | – | – | – | – | – | – | – | – |
| Private (purchased) | 0.267 (−0.081, 0.614) | 0.133 | 0.242 (−0.057, 0.542) | 0.113 | −0.025 (−0.320, 0.269) | 0.866 | 0.049 (−0.249, 0.346) | 0.748 |
| Renting | 0.605 (−0.097, 1.308) | 0.091 | 0.398 (−0.231, 1.027) | 0.215 | 0.043 (−0.573, 0.659) | 0.892 | 0.200 (−0.397, 0.797) | 0.511 |
| Living arrangement | | | | | | | | |
| Living with others † | – | – | – | – | – | – | – | – |
| Living alone | 0.150 (−0.267, 0.567) | 0.480 | −0.069 (−0.448, 0.309) | 0.719 | −0.176 (−0.545, 0.194) | 0.351 | −0.189 (−0.542, 0.165) | 0.296 |
| Household with car | | | | | | | | |
| No † | – | – | – | – | – | – | – | – |
| Yes | −0.043 (−0.381, 0.295) | 0.802 | 0.107 (−0.198, 0.412) | 0.490 | −0.043 (−0.341, 0.255) | 0.776 | 0.298 (0.011, 0.585) * | 0.042 |
| Area-level socio-economic status | | | | | | | | |

| | | | | | | | | |
|---|---|---|---|---|---|---|---|---|
| Low† | – | – | – | – | – | – | – | – |
| High | 0.019 (−0.355, 0.394) | 0.919 | −0.082 (−0.368, 0.204) | 0.575 | −0.082 (−0.367, 0.203) | 0.573 | 0.112 (−0.216, 0.439) | 0.504 |
| Recruitment area | | | | | | | | |
| Community centre † | – | – | – | – | – | – | – | – |
| Elderly health centre | −0.045 (−0.459, 0.369) | 0.830 | −0.182 (−0.520, 0.157) | 0.292 | −0.205 (−0.540, 0.130) | 0.230 | −0.562 (−0.919, −0.205) ** | 0.002 |
| Number of current diagnosed health problems | −0.329 (−0.409, −0.294) *** | <0.001 | −0.177 (−0.249, −0.104) *** | <0.001 | −0.072 (−0.143, −0.002) * | 0.045 | −0.033 (−0.101, 0.035) | 0.338 |

Notes: b = regression coefficient; CI = confidence interval; *p* = *p* value; - = not applicable. † reference group. * p < 0.05; ** p < 0.01; *** p < 0.001.

**Table S3.** Associations between single GIS neighbourhood environmental attributes based on 400 m street-network buffers and quality of life (QoL) domains.

| Environmental Attributes | (Unit) | Physical QoL | | Psychological QoL | | Social QoL | | Environmental QoL | |
|---|---|---|---|---|---|---|---|---|---|
| | | ***b* (95% *CI*)** | ***p*** | ***b* (95% *CI*)** | ***p*** | ***b* (95% *CI*)** | ***p*** | ***b* (95% *CI*)** | ***p*** |
| Gross residential density | (1000 households/km²) | −0.009 (−0.025, 0.007) | 0.262 | 0.006 (−0.007, 0.018) | 0.396 | −0.005 (−0.018, 0.008) | 0.443 | −0.008 (−0.022, 0.006) | 0.248 |
| Street intersection density | (100 intersections/km²) | 0.089 (−0.197, 0.374) | 0.542 | −0.208 (-0.451, 0.034) | 0.093 | −0.132 (−0.370, 0.105) | 0.274 | −0.277 (−0.517, −0.037) * | 0.024 |
| Civic and institutional density | (destinations/km²) | −0.001(−0.004, 0.002) | 0.585 | −0.000 (−0.003, 0.002) | 0.861 | −0.001 (−0.004, 0.001) | 0.312 | −0.001 (−0.004, 0.002) | 0.445 |
| Entertainment density | (destinations/km²) | 0.001(−0.010, 0.011) | 0.907 | 0.001(−0.007, 0.010) | 0.786 | −0.006 (−0.014, 0.003) | 0.181 | -0.007 (−0.016, 0.002) | 0.132 |
| Recreation density | (destinations/km²) | −0.001 (−0.007, 0.006) | 0.879 | 0.001 (−0.005, 0.007) | 0.762 | 0.005 (−0.001, 0.010) | 0.128 | 0.003 (−0.002, 0.009) | 0.255 |
| Park area | (hectares) | −0.013 (−0.029, 0.003) | 0.112 | 0.004 (−0.010, 0.018) | 0.627 | −0.008 (−0.022, 0.007) | 0.305 | −0.001 (−0.015, 0.013) | 0.882 |

*Notes: b* = regression coefficient; *CI* = confidence interval; *p* = *p* value; - = not applicable; GIS = geographic information systems; All estimates adjusted for age, sex, educational attainment, household with car, marital status, housing type, living arrangement, area-level socio-economic status, type of recruitment centre, and number of current diagnosed health problems. "0.000" occurs due to rounding and does not equal to zero. * *p* < 0.05.
